# Identification of shared key genes in Rheumatoid Arthritis and COVID-19 and their relevance as diagnostic biomarkers and with immune infiltration: New insights from bioinformatics analysis

**DOI:** 10.1371/journal.pone.0333886

**Published:** 2025-10-09

**Authors:** Wei Ya Lan, Shan Shan Cai, QianWei Lu, Fang Tang

**Affiliations:** 1 Graduate School, Guizhou University of Traditional Chinese Medicine, Guiyang, Guizhou, China; 2 Department of Joint and Orthopedic Surgery, Second Affiliated Hospital of Guizhou University of Traditional Chinese Medicine, Guiyang, Guizhou, China; 3 Department of Rheumatology and Immunology, Second Affiliated Hospital of Guizhou University of Traditional Chinese Medicine, Guiyang, Guizhou, China; Nanjing First Hospital, Nanjing Medical University, CHINA

## Abstract

The interaction mechanism between Coronavirus Disease (COVID-19) and rheumatoid arthritis (RA) remains inadequately understood. Consequently, this study sought to elucidate the potential mechanisms underlying the comorbidity between RA and COVID-19, as well as to identify key genes, diagnostic markers, and associated immune cells. Differential analysis of the training set, derived from the GEO database, identified differentially expressed genes (DEGs) in the RA and COVID-19 gene chip and sequencing datasets. Weighted Gene Co-expression Network Analysis (WGCNA) identified key modular genes, while protein-protein interaction (PPI) network analysis revealed hub genes, which were validated by the validation set. Receiver Operating Characteristic (ROC) curves were used to assess clinical relevance. Cytoscape-based transcription factor (TF)–mRNA and microRNA (miRNA)–mRNA regulatory networks were used to identify potential therapeutic targets, and immune cell infiltration was evaluated using the CIBERSORT algorithm. Differential expression analysis identified 2,778 DEGs in RA and 12,733 in COVID-19, with WGCNA identifying 18 shared genes, suggesting possible common molecular mechanisms. Validation analysis confirmed *LGMN* and *NRGN* as key genes associated with RA and COVID-19 comorbidity, highlighting their diagnostic significance. Network analysis identified related miRNAs and TFs, and enrichment analysis revealed the critical signaling pathways. Immune cell infiltration in patients with RA and COVID-19 was assessed using the CIBERSORT algorithm. This study preliminarily explored the shared pathogenic mechanisms between RA and COVID-19, identifying *LGMN* and *NRGN* as potential biomarkers for both diseases. Notably, *NRGN* may play a significant role as a common biomarker involved in the immune response in both disease states. These findings may open new avenues for the diagnosis and treatment of RA and COVID-19.

## Introduction

Rheumatoid arthritis (RA) is an autoimmune disease that primarily affects multiple joints, resulting in irreversible cartilage and bone damage and disability [1]. Its pathology is characterized by chronic synovial membrane inflammation, cartilage destruction, autoimmune antibody production, and multiorgan lesions [2,3]. The disease has a complex pathogenesis, and treatment typically involves nonsteroidal anti-inflammatory drugs, disease-modifying antirheumatic drugs, and glucocorticoids, which alleviate symptoms but have adverse effects with long-term use [4]. Research on RA from various perspectives has offered diverse therapeutic possibilities [5,6]. There is a significant association between viral infections and arthritis, with respiratory viruses often causing joint pain [7]. Evidence links respiratory viral infections to the development of RA [8], and these infections can trigger inflammatory arthritis and acute flares [9].

Coronavirus Disease (COVID-19) is caused by a severe acute respiratory syndrome (SARS) related coronavirus strain [10,11]. The pandemic has severely impacted global health and the economy. Research has indicated that the virus triggers an excessive inflammatory response, leading to a cytokine storm and severe COVID-19, suggesting that immunosuppression may be beneficial [12]. There may be a significant link between the clinical manifestations and immune responses to COVID-19 and other autoimmune diseases [13]. Notably, some diagnostic and therapeutic strategies for rheumatoid arthritis (RA) are also applicable to COVID-19 [14]. Individuals with autoimmune diseases, such as RA, are at a higher risk of COVID-19 infection and severe disease, with evidence of infection being common [15,16]. The common features of RA and COVID-19 include elevated pro-inflammatory cytokine levels, lymphopenia, neutrophil and macrophage activation, and shared immune pathways [17]. Considering the similarities in susceptibility and aberrant immunoinflammatory activity between RA and COVID-19, key genes and common genetic structures for gene expression may be present.

COVID-19 pathogenesis resembles that of autoimmune diseases, in which autoantibodies are common [18]. This virus directly and indirectly affects multiple organs, including the musculoskeletal system. Skeletal muscle damage involves muscle fiber atrophy, sporadic myofibrillar necrosis, and immune cell infiltration [19]. COVID-19 frequently causes arthralgia and reduced bone marrow density, with an increased risk of osteoporosis following glucocorticoid use [20]. RA can also lead to osteoporosis, and biologics may reduce this risk [21,22]. Clinical reports have noted instances of RA after post-COVID-19 [23,24]. This study aimed to identify the key genes common to RA and COVID-19, assess their diagnostic potential, and explore their pathogenesis.

## Materials and methods

### Data source

Gene expression profiles were obtained from the Gene Expression Omnibus (GEO) database using MeSH Heading terms “Arthritis, Rheumatoid” and “COVID-19.” The RA training set included GSE77298 (seven normal and 16 RA synovial) and GSE206848 (seven normal, seven osteoarthritis (OA), and two RA synovial samples). Patients with OA were excluded from this study. The COVID-19 training set used GSE164805 (five normal, five mild, and five severe cases). GSE55457 served as the RA validation set, and GSE171110 as the COVID-19 validation set. The data were normalized, invalid samples were removed, and de-batching was performed for multiple datasets before analysis. Table 1 details the dataset information, including the microarray platforms, sample sets, and quantities.

**Table 1:** Basic information on the datasets used in this study.

Datasets	Expression profiling	Sample size		Platform
		Normal	RA	
GSE77298	array	7	16	GPL570
GSE206848	array	7	2	GPL570
GSE55457	array	10	13	GPL96
		Normal	COVID-19	
GSE164805	array	5	10	GPL26963
GSE171110	high throughput sequencing	10	44	GPL16791

For rheumatoid arthritis (RA), the GSE77298 and GSE206848 datasets were combined to form the training set, and GSE5547 was used for external validation. For COVID-19, GSE164805 was used as the training dataset and GSE171110 as the validation set.

### Differential expression analysis

Differential expression analysis between the disease and normal groups in both the RA and COVID-19 datasets was performed using the “limma” package (version 3.52.2). Differentially expressed genes (DEGs) were identified based on the criteria of |log2FC| > 1 and adjusted P < 0.05. Accordingly, DEGs were obtained for each disease. To control for false discoveries due to multiple testing, P-values were adjusted using the Benjamini-Hochberg method, with an adjusted P-value (FDR) of < 0.05 considered statistically significant.

### Screening the characteristic genes through weighted gene co-expression network analysis (WGCNA)

WGCNA was performed on the DEGs of each disease group. Sample clustering showed good consistency, with a cut height set at 20,000 and a soft-thresholding power range of 1:20, which was used for network topology analysis. Module Membership (MM) and Gene Significance (GS) were calculated to assess gene–module and gene–trait correlations, respectively, and visualized using scatter plots. Pearson’s correlation analysis was used to estimate the association between module eigengenes and disease status. The most disease-relevant modules were selected, and their intersecting genes were extracted for further analyses.

### Functional enrichment, PPI network construction, and validation using independent dataset and confidence intervals

The biological roles of shared modular genes in RA and COVID-19 were visualized by converting the molecular lists to IDs using “clusterProfiler” (version 4.4.4) and performing Gene Ontology (GO) and Kyoto Encyclopedia of Genes and Genomes (KEGG) enrichment analyses (corrected P-value < 0.05). To investigate potential interactions among the candidate pathogenic proteins, a protein–protein interaction (PPI) network was constructed using STRING database (https://string-db.org/). Protein names were input with the species set to *Homo sapiens*, and interactions were retrieved based on both known and predicted associations with a confidence score threshold of > 0.04. The resulting PPI network was visualized using Cytoscape (version 3.10.0). Key hub proteins were identified using the cytoHubba plugin, and node ranking was performed based on the Maximal Clique Centrality (MCC) algorithm.

For the common hub genes identified in the validation sets of both diseases, the log fold change (logFC) between the disease and control groups was calculated, and 95% confidence intervals (CIs) were derived using the formula: CI=logFC±1.96×SE . The results were reported as CI = [CI.lower, CI.upper], indicating a 95% probability that the true log₂ expression difference (RA vs. control) in the overall population lies within this interval.

### Assessing the diagnostic efficacy of key genes in disease

Core genes were screened from the validation sets of the two diseases for expression level analysis, and if they were all significant in the validation sets, they were regarded as key genes. Subsequently, the diagnostic value of the core genes in the clinical setting was determined using the ROC curve and evaluated using the Area Under Curve (AUC). The closer the AUC is to 1, the better the diagnostic effect of the variable in predicting the outcome in the case of AUC > 0.5. ROC analysis of the data was performed using the pROC (1.18.0) package, and the results were visualized using the ggplot2 (3.3.6) package.

### Constructing gene regulatory networks and predicting interactions between transcription factors, miRNAs and key genes

Transcription factors (TFs) and miRNAs associated with key genes were predicted using NetworkAnalyst (https://www.networkanalyst.ca/NetworkAnalyst). Data for transcription factors and genes were sourced from the ENCODE ChIP-seq database. The criteria included peak intensity signals <500 and regulatory potential scores <1 (BETA Minus algorithm). The experimentally validated miRNA-gene interaction data were obtained from miRTarBase. A topological network of TFs and miRNAs was constructed using the Cytoscape software. KEGG analysis was performed to identify relevant pathways.

### Immune infiltration analysis

Immune infiltration was assessed using the CIBERSORT algorithm with the CIBERSORT (v1.03) R package and markers for 22 immune cells from the CIBERSORTx website (https://cibersortx.stanford.edu/) [25]. The proportions of these cells in each sample are displayed as stacked bar graphs. The expression levels between the normal and diseased groups and the immune-infiltrating cells were compared. Correlation heatmaps were used to illustrate the relationships between immune cells. Finally, a lollipop graph was used to visualize the correlation between the two key genes and various immune-infiltrating cells. Graphs were created using the ggplot2 package.

### Drug repositioning analysis for hub genes

To identify potential drug candidates for the validated hub genes, we performed a drug repositioning analysis using the Drug Signatures Database (DSigDB) available on the Enrichr platform (https://maayanlab.cloud/Enrichr/) [26–28]. A list of shared hub genes identified in both RA and COVID-19 was uploaded to the Enrichr tool. DSigDB was selected as the reference database, which outputs a ranked list of compounds predicted to interact with input genes. These drugs may modulate the common molecular pathways involved in both RA and COVID-19, suggesting their potential therapeutic relevance.

### Statistical analysis

Statistical analyses were performed using R software (version 4.2.1). The stats (4.2.1) and car (3.1−0) packages were used to select suitable statistical methods based on the data characteristics, and the ggplot2 package was used for data visualization. Spearman’s correlation analysis was used to estimate the correlation coefficients between genes, with statistical significance set at P < 0.05.

## Results

### The RA and COVID-19 datasets were screened separately for DEGs

**Fig 1:**
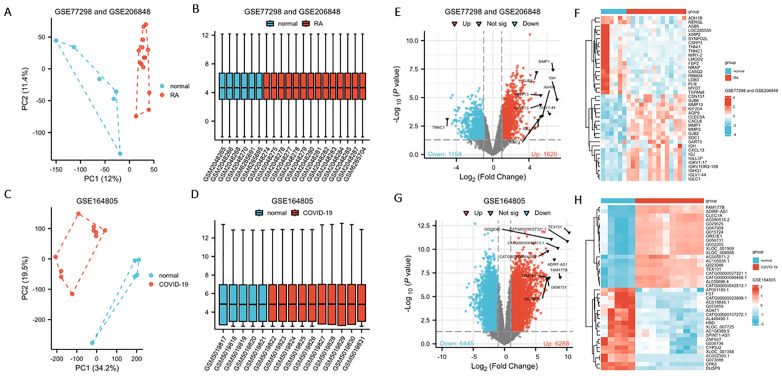
Differentially expressed genes (DEGs) identified between the normal and disease groups in the training datasets for rheumatoid arthritis (RA) and COVID-19. “Normal” refers to the control group. (A, B) PCA plot and normalized box plot of merged RA datasets. (C, D) PCA and box plots of the COVID-19 dataset. (E, F) Volcano plot and heatmap showing the DEGs in RA. (G, H) Volcano plot and heatmap showing the DEGs in COVID-19.

The RA dataset combined the GSE77298 and GSE206848 datasets as the training set. After downscaling high-dimensional data using PCA plots to standardize the data and remove invalid overlapping samples, inter-sample differences between the normal and disease groups were evaluated. Box plots ensured the median of each sample was consistent (Fig 1A and 1B). Differential analysis identified 2778 DEGs, including 1620 upregulated and 1158 downregulated genes. In addition, the top 10 most significantly differentially expressed genes were labeled in the volcano plot, and heatmaps illustrated the top 20 upregulated and downregulated genes in each dataset (Fig 1E and 1F). GSE164805 was used as the training dataset for COVID-19 analysis. After data preprocessing, data quality was visualized using PCA and box plots (Fig 1C and 1D). Differential expression analysis identified 12,734 DEGs, including 6,288 upregulated and 6,445 downregulated genes. The filtered DEGs were visualized using volcano and heat maps (Fig 1G and 1H) (S1 and S2 Tables).

### Analysis of the co-expressed genes in RA and COVID-19

**Fig 2:**
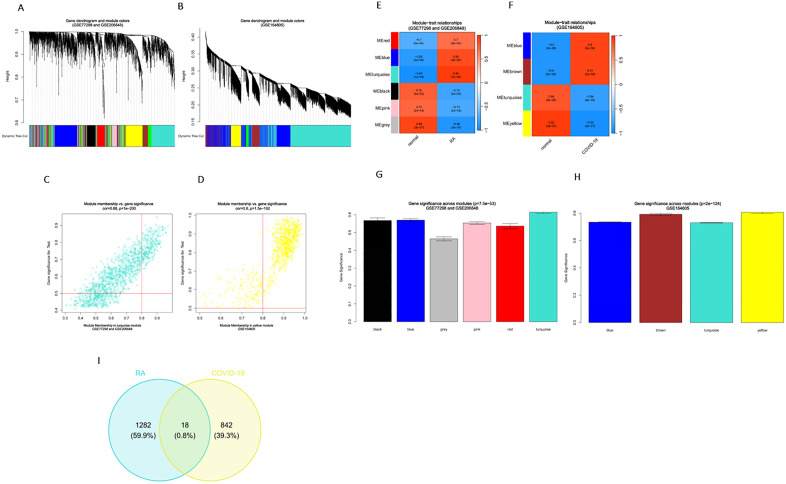
WGCNA of DEGs in RA and COVID-19. (A) Cluster dendrogram of RA DEGs based on topological overlap, with assigned module colors and module eigenvalues. (B) Cluster dendrogram of COVID-19 DEGs in. (C) Module–trait relationship heatmap for RA. The module colors are shown on the left, and the correlation strength is represented on the right. In the heatmap, red indicates a strong positive correlation and blue indicates a negative correlation. (D) Module–trait heatmap for COVID-19. (E) Scatter plot showing the correlation between Module Membership (MM) and Gene Significance (GS) in the turquoise module of RA. (F) Scatter plot of the yellow module in COVID-19. (G) Bar plot showing module significance in RA, with taller bars indicating higher significance of the module. (H) Bar plot of module significance in COVID-19. (I) Venn diagram showing 18 overlapping genes between the most significant modules of RA and COVID-19.

Weighted gene co-expression network analysis (WGCNA) was performed to identify gene modules most strongly associated with RA and COVID-19 based on the respective DEGs. Genes with similar expression patterns were clustered into modules using Spearman’s correlation coefficients. In the RA training set (GSE77298 and GSE206848), six modules were identified, with the turquoise module showing the strongest positive correlation with RA (correlation coefficient = 0.94, p = 1e − 9), comprising 1,300 genes (Fig 2A and 2C). In the COVID-19 training set (GSE164805), four modules were identified, with the yellow module showing the strongest negative correlation with disease status (correlation coefficient = −0.92, p = 9e − 7), and containing 860 genes (Fig 2B and 2D). Scatter plots illustrated the correlation between module membership (MM) and gene significance (GS) in the turquoise module of RA and the yellow module of COVID-19 (Fig 2E and 2F, respectively). Bar plots of module–trait relationships showed that these two modules had the highest correlation scores in each dataset (Fig 2G and 2H, respectively). Finally, a Venn diagram revealed 18 overlapping genes shared between the RA- and COVID-19-associated modules (Fig 2I).

### Construction of Protein–Protein Interaction (PPI) network, screening of hub genes and exploration of potential biological functions

**Fig 3:**
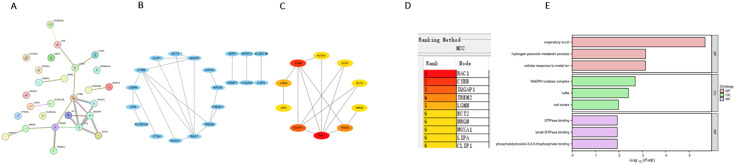
Identification and functional analysis of hub genes. (A) Protein–protein interaction (PPI) network constructed using the STRING database with a confidence score > 0.04. (B) PPI network visualized using Cytoscape software. (C, D) Top 10 hub genes identified using the Maximal Clique Centrality (MCC) algorithm; darker colors indicate higher centrality within the PPI network. (E) GO enrichment analysis of shared candidate genes, showing the top three terms for biological process (BP), cellular component (CC), and molecular function (MF).

To identify key diagnostic candidate genes for both RA and COVID-19, the 18 overlapping DEGs were input into the STRING database to construct a protein–protein interaction (PPI) network. To preserve the full scope of protein interactions, isolated nodes were retained for network visualization (Fig 3A). Within the PPI network, *CYBB* and *RAC1* were identified as the core nodes. *IQGAP1* and *TREM2* interacted with at least four proteins. Notably, *LGMN* directly interacted with *CYBB*, one of the core nodes, while *NRGN* directly interacted with *TREM2* and may be indirectly linked to *RAC1*.

To further validate and rank the key genes within the PPI network, we applied the Maximal Clique Centrality (MCC) algorithm in Cytoscape. The top 10 genes, based on the MCC ranking, were selected as candidate diagnostic biomarkers (Fig 3B–3D). To elucidate the biological functions and pathways associated with these shared candidate genes, Gene Ontology (GO) and Kyoto Encyclopedia of Genes and Genomes (KEGG) enrichment analyses were performed. A total of 187 significantly enriched GO terms (FDR < 0.05) were identified, including 135 biological processes (BP), 24 cellular components (CC), and 28 molecular functions (MF). Representative GO terms, including 3 BP, 3 CC, and 3 MF terms, are shown in Fig 3E. No KEGG pathways reached statistical significance after multiple testing corrections.

In the BP category, genes were primarily involved in respiratory burst, hydrogen peroxide metabolic processes, |and cellular responses to metal ions. In the CC category, enrichment was observed in the NADPH oxidase complex, ruffles, and cell cortex. For MF, the enriched terms included GTPase binding, small GTPase binding, and phosphatidylinositol-3,4,5-trisphosphate binding (S3 Table).

### Validation of hub gene differential expression and robustness assessment via confidence intervals

**Fig 4:**
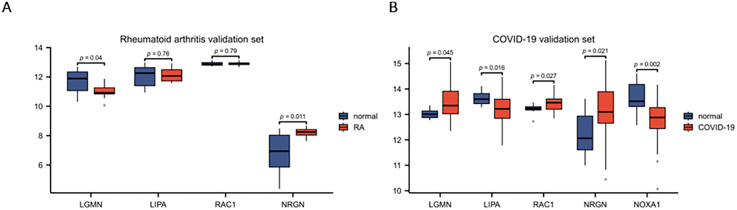
Comparison of gene expression between normal and disease groups. (A, B) Differentially expressed genes were selected (P < 0.05) using the normal group as a reference. Genes positioned above the central horizontal line represent upregulation, whereas those below indicate downregulation in the disease group.

The GSE171110 dataset was used as a COVID-19 validation set, which verified that *LGMN*, *LIPA*, *RAC1*, *NRGN*, and *NOXA1* were significantly different (P < 0.05) between the normal and disease groups, with *LGMN*, *RAC1*, and *NRGN* being highly expressed relative to the normal group (Fig 4A). In contrast, the GSE55457 dataset, which served as the RA validation set, only validated that *LGMN* and *NRGN* were significantly different between the normal and disease groups (P < 0.05), where *NOXA1* was not found, and *NRGN* was highly expressed relative to the normal group (Fig 4B). In addition, 95% confidence intervals (CIs) were calculated for the shared hub genes to validate the direction and precision of the differential expression in both diseases, further demonstrating the robustness of the findings (Table 2).

**Table 2:** Differential expression and 95% confidence intervals of shared hub genes in the RA and COVID-19 validation datasets.

Datasets	Genes	logFC	P	FDR	95%CI
GSE55457	***LGMN***	−0.689	0.023	0.046	**(−1.241, −0.137)**
	*LIPA*	0.085	0.744	0.813	(−0.416, −0.0.585)
	*RAC1*	0.015	0.813	0.813	(−0.107, 0.137)
	***NRGN***	1.451	0.002	0.008	**(0.638, 2.264)**
GSE171110	***LGMN***	0.410	0.049	0.049	**(0.012, 0.809)**
	*LIPA*	−0.423	0.033	0.046	(−0.802, −0.044)
	*RAC1*	0.227	0.034	0.046	(0.022, 0.432)
	***NRGN***	0.909	0.017	0.046	**(0.186, 1.632)**

Differential expression analysis of the four shared hub genes (LGMN, LIPA, RAC1, and NRGN) was performed using the RA validation dataset (GSE55457) and the COVID-19 validation dataset (GSE171110). The columns indicate the log fold change (logFC), p-value, false discovery rate (FDR), and 95% confidence interval (CI) for each gene. Genes with FDR < 0.05 in both datasets are shown in bold, indicating statistically significant differential expression in both diseases.

### Analysis of the diagnostic value of key genes in two diseases

**Fig 5:**
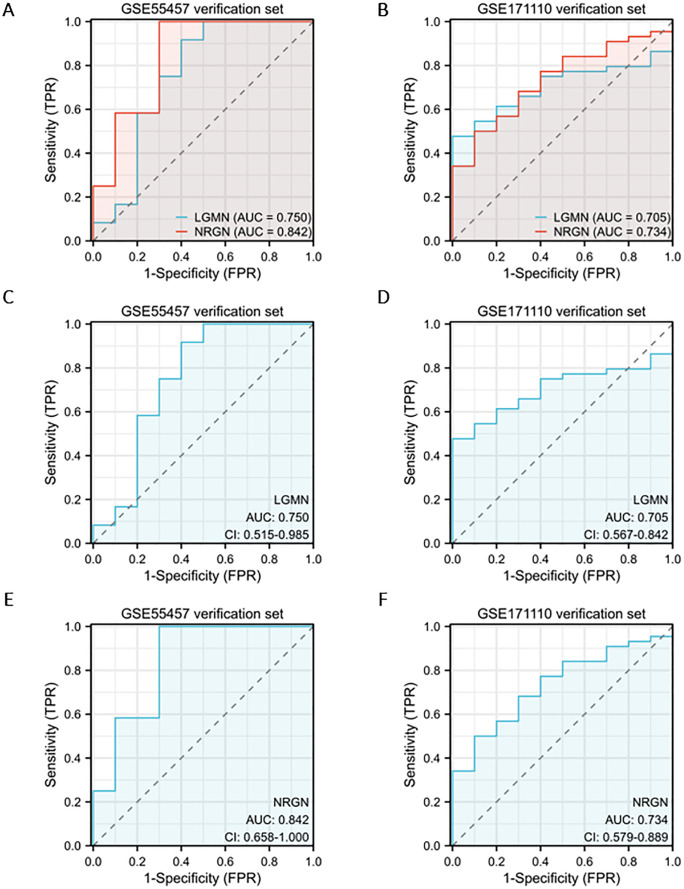
Diagnostic value assessment of the validation group (GSE55457 and GSE171110). (A, B) ROC plots for key genes (*LGMN* and *NRGN*) based on AUC. (C-F) ROC plots with AUC and CI of individual genes in the corresponding diseases. GSE55457: rheumatoid arthritis dataset. GSE171110: COVID-19 dataset. AUC: area under the curve. CI: 95% confidence interval.

The diagnostic value of the key genes in the two diseases was evaluated separately using ROC curves. The results indicated that in the validation set for COVID-19, GSE171110 had an AUC of 0.705 (0.567–0.842) for *LGMN* and 0.734 (0.579–0.889) for *NRGN* (Fig 5B, 5D, and 5F). In the RA validation set GSE55457, the AUC values were 0.750 (0.515–0.985) for *LGMN* and 0.842 (0.658–1.000) for *NRGN* (Fig 5A, 5C and 5E). According to the AUC assessment criteria, an AUC of 0.5–0.7 indicates low accuracy, 0.7–0.9 indicates moderate accuracy, and above 0.9 indicates high accuracy. These ROC results suggest that LGMN and NRGN are effective diagnostic markers for predicting RA and COVID-19 (S4 Table).

### Identification and functional analysis of transcription factors and miRNAs associated with *LGMN* and *NRGN*

**Fig 6:**
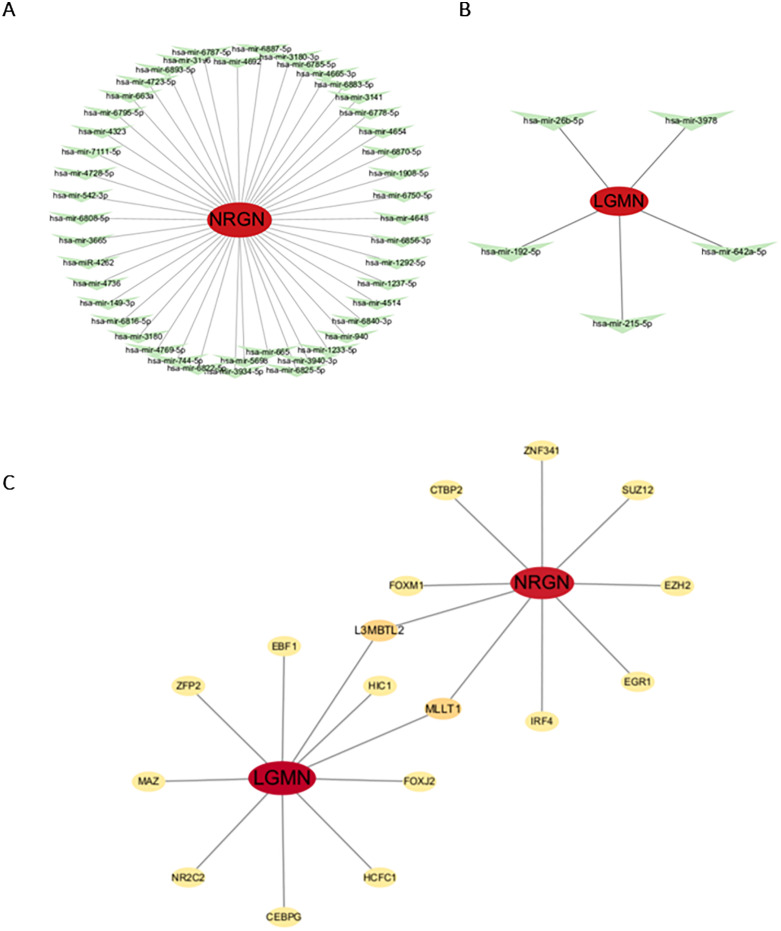
miRNA and transcription factor regulatory networks of *NRGN* and *LGMN.* (A, B) miRNA–mRNA interaction networks related to *NRGN* and *LGMN*. (C) TF–mRNA regulatory network. Red circles represent key genes, green inverted triangles indicate miRNAs, and yellow circles represent transcription factors (TFs).

Using the miRTarBase database, 45 miRNAs were identified as being associated with *LGMN* and five with *NRGN*. The network constructed with *LGMN* and miRNAs comprised 46 nodes and 45 edges, whereas the NRGN network included six nodes and five edges (Fig 6A and 6B). ENCODE ChIP-seq data revealed that 10 TFs were linked to *LGMN* and nine to *NRGN*, with L3MBTL2 and MLLT1 being common to both (Fig 6C). NetworkAnalys identified transcription factor-enriched pathways common to both key genes, as well as those individually predicted for each gene and their shared transcription factors. KEGG analysis indicated significant involvement of antigen processing and presentation, lysosomes, and transcriptional misregulation in cancer.

### Immune Infiltration Analysis

**Fig 7:**
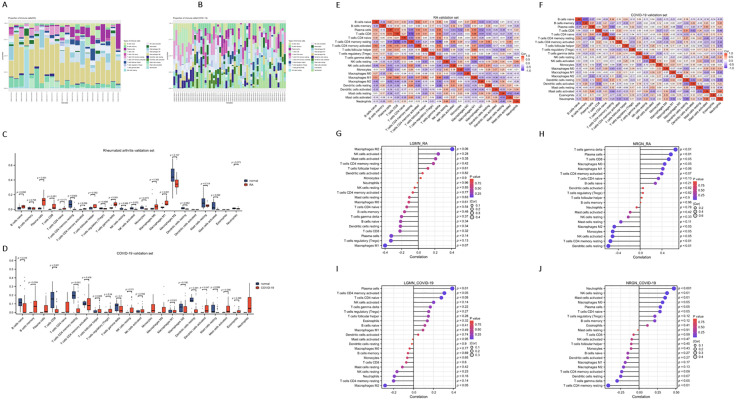
Immune cell infiltration analysis in the RA and COVID-19 validation datasets. (A, B) The relative abundance of immune cell subtypes in the normal and disease groups is presented, with the cell types labeled on the right. Eosinophils are not shown in panel A, as their inferred relative abundance was zero in both normal and RA samples. (C, D) Comparison of immune cell proportions between the disease and control groups. Statistical tests were not conducted for subgroups with fewer than three samples or with a standard deviation of zero; p-values were omitted in these cases. (E, F) Correlation heatmap of the immune cell subpopulations. (G-J) Correlation between *LGMN* and *NRGN* expression and infiltrating immune cells in patients with RA and COVID-19.

Both RA and COVID-19 are closely associated with the human immune system. Therefore, we employed the CIBERSORT algorithm to compare the infiltration levels of 22 immune cell types between disease and normal samples in RA and COVID-19. The analysis revealed significant differences in the activation of multiple immune cell populations (S5 and S6 Tables). Key genes and immune-infiltrating cells were examined in both diseases. Stacked bar graphs depict the ratio of immune cells (Fig 7A and 7B), with five and seven cell types showing significant differences (P < 0.05) in patients with RA and COVID-19, respectively (Fig 7C and 7D). The varying degrees of immune cell infiltration in both diseases may serve as therapeutic targets. Spearman correlation coefficients showed a significant negative correlation (r = −0.72) between T cells CD4 memory resting and T cells CD8 in RA (Fig 7E) and between memory B cells and naïve B cells in COVID-19 (Fig 7F).Correlation analysis of diagnostic biomarkers with immune cell patterns showed that in RA, NRGN was positively correlated with plasma cells (r = 0.56, P = 0.007), T cells CD8 (r = 0.52, P = 0.012), gamma delta T cells (r = 0.65, P = 0.001), and Macrophages M0 (r = 0.45, P = 0.036), and negatively correlated with CD4 memory resting T cells (r = −0.54, P = 0.009), activated NK cells (r = −0.51, P = 0.016), monocytes (r = −0.50, P = 0.017), Macrophages M2 (r = −0.47, P = 0.027), and resting dendritic cells (r = −0.58, P = 0.004) (Fig 7G and 7H). In COVID-19, *LGMN* was positively correlated with plasma cells (r = 0.39, P = 0.004), T cells CD4 memory activated (r = 0.31, P = 0.023), and T cells CD4 naive (r = 0.28, P = 0.041), and negatively with Macrophages M2 (r = −0.29, P = 0.036). *NRGN* was linked to neutrophils (r = 0.47, P < 0.001), resting NK cells (r = 0.38, P = 0.004), activated mast cells (r = 0.35, P = 0.009), Macrophages M0 (r = 0.32, P = 0.018), and plasma cells (r = 0.29, P = 0.034), and negatively with T cells CD4 memory resting (r = −0.42, P = 0.002) and T cell gamma delta (r = −0.30, P = 0.028) (Fig 7I and 7J).

### Drugability assessment of hub genes

We used the Drug Signatures Database (DSigDB) within the Enrichr open-source gene set enrichment platform to identify potential drug candidates targeting the validated hub genes LGMN and NRGN. The results are presented in Table 3 (see S7 Table for details).

**Table 3:** Candidate drug predicted using DSigDB.

Drug names	P-value	Adjusted P-value	Genes
trichostatin A PC3 UP	0.0004	0.0137	NRGN;LGMN
trichostatin A MCF7 UP	0.0019	0.0305	NRGN;LGMN
cimetidine BOSS	0.0031	0.0328	LGMN
asparagine BOSS	0.0041	0.0328	LGMN

Only results with statistically significant adjusted p-values (FDR < 0.05) are presented in this table. Each gene represents a predicted target of the corresponding drug(s); multiple drugs targeting the same gene are separated for clarity.

## Discussion

Most of the collated articles indicated that the manifestations of various autoimmune or rheumatic diseases are associated with COVID-19 and may be the triggering etiology [16].The relationship between COVID-19 and viral arthritis was reported in 2020, and the analysis of synovial tissue with validation of the response after viral infection is particularly important [29].RA is an invasive disease caused by inflammation of the synovial lining and destruction of cartilage. Chronic inflammatory joint disease due to causative factors may be environmental risk factors, such as respiratory viral infections, which are associated with an increase in the number of RA cases [8]. According to the COVID-19 Global Alliance for Rheumatic Diseases (GRA) registry, approximately 40% of the total number of COVID-19 cases have RA [30]. In particular, during the COVID-19 pandemic, patients with RA were more likely to develop infections [31]. In this study, to determine the relationship and mechanism of action between RA and COVID-19, differentially expressed genes (DEGs) were identified by differential analysis from the perspective that there may be common key genes between them, and the presence of up- and down-regulated genes in each was found. However, we were not satisfied with this, and to identify clusters (modules) of highly related genes in each disease, the DEGs of each disease were clustered into different modules based on similarity using the weighted gene co-expression network analysis (WGCNA) method [32].

In this study, a comorbidity model of RA and COVID-19 was constructed, and hub genes between the diseases, including *RAC1*, *CYBB*, *IQGAP1*, *TREM2*, *LGMN*, *ECT2*, *NRGN*, *NOXA1*, *LIPA*, and *CLIP1*, were obtained using two progressive genetic screens. Enrichment analysis showed that “ cellular response to metal ion,” “respiratory burst” and “hydrogen peroxide metabolic process “ in biological processes (BPs) may play important roles in RA and COVID-19. Recent studies have identified three traits of COVID-19 (severe COVID-19, COVID-19 hospitalization, and SARS-CoV-2 infection) and determined that loci potentially causally and co-associated with RA were significantly enriched in the spleen, lungs, whole blood, and small intestine [33]. In our study, COVID-19 and RA were divided into normal and disease groups only, with whole blood samples from COVID-19 patients and synovial samples from patients with RA. To validate the RA and COVID-19 training set results, we chose the validation sets RA as GSE55457 [34] and GSE171110 [35]. *LGMN* and *NRGN* were identified as significantly expressed in the validation set.

Legumain (*LGMN*), also known as putative cysteine protease (*PRSC1*), belongs to the peptidase C13 family, and its subcellular location of its mature protein is located in the lysosome, which is involved in protein processing for MHC class II antigen presentation in the lysosome/endosomal system [36]. In the zymogen form, the uncleaved prepeptide blocks access to the active site [37]. Its tissue specificity is enriched in the kidneys, heart, and placenta [38]. According to the Bgee gene expression database [39], this gene is most highly expressed in the synovial joints. One study explored the presence of this gene in COVID-19 patients. By distinguishing the cell surface proteins of transcriptional subpopulations, it was found that a higher percentage of CD163/*LGMN* alveolar macrophages was associated with mortality due to acute hypoxic respiratory failure [40]. In another study, a close correlation between *LMGN* and alveolar macrophages was reported [41]. In summary, we found that *LGMN* is associated with alveolar macrophages and synovial joints. There is evidence that macrophages present in bronchial and synovial tissues are heterogeneous; however, bronchoalveolar lavage macrophage clusters in COVID-19 have functional characteristics similar to those of synovial tissue macrophage clusters in synovitis in RA, including transcriptional and functional homology in healthy individuals [42]. Thus, RA and COVID-19 may share a common immunopathogenic pathway driven by the activity of similar macrophage clusters. The ROC analysis in our study also demonstrated the diagnostic value of *LGMN* in patients with RA and COVID-19. However, the relationship between *LGMN* and RA in the current study is unclear, and it may also be due to its important role only in comorbidities with COVID-19.

Neurogranin (*NRGN*) is a neural granule protein with a subcellular location of the mature protein in the cytoplasm, based on the selection of gene ontology (GO) terms. Calmodulin (CaM) may be the only major protein interacting with neural granule proteins, and may also regulate its own binding to CaM by phosphorylation at the Ser-36 site, acting as a protein kinase C-mediated “third-messenger” substrate during salient developmental and remodeling processes, and playing a role in neural system development and signal transduction [43]. Recent studies have pointed to a potential pathogenesis between COVID-19 and Alzheimer’s disease (AD), and *NRGN* has been proposed as a shared molecule [44,45]. A complex link exists between their shared pathophysiological mechanisms, which involve immune dysregulation [46]. In addition, due to the chronic inflammation of RA with AD, the extracts mentioned in studies on the treatment of RA are effective in the treatment of AD, and a variety of diseases, including them, may have potential therapeutic targets [47,48]. There is a strong negative correlation between RA and AD risk when engaging in sedentary behavior due to computer use during leisure time [49]. There is also a potential causal relationship between RA and other neurodegenerative diseases [50]. However, *NRGN* has not emerged as a relevant study for RA. In the present study, *NRGN* was identified as a key gene associated with both RA and COVID-19. ROC curve analysis further supported its potential diagnostic value, suggesting its role as a shared molecular biomarker between these two diseases. We speculated that RA and COVID-19 may share a common role in affecting the nervous system and its signaling.

Subsequently, we constructed regulatory networks with transcription factors (TFs) and miRNAs based on the identified key genes to explore their corresponding biological mechanisms. KEGG prediction analysis using common transcription factors related to *LGMN* and *NRGN* showed significant enrichment in the pathways of antigen processing and presentation, lysosomes, and transcriptional misregulation in cancer. This implies that there may be potential pathways that influence the development of both RA and COVID-19. In immunology, RA is characterized by dysregulation of innate immune responses, adaptive immunity targeting self-antigens, and cytokine imbalance. Throughout disease progression, infiltrating immune cells in the joints and the presence of circulating autoantibodies play a central role [51]. New evidence suggests that Th cells play a major role in the pathogenesis of RA, with overactivation of Th1 cells triggering the secretion of proinflammatory mediators and recruitment of macrophages as antigen-presenting cells, while Th2 cells also play a key role in the differentiation of lymphocytes to produce IgE antibodies [52–54]. This is also in line with our results, which suggested by RA immune infiltration analysis that Th1 and Th2 are expressed in the disease, but there is no corresponding expression in COVID-19. This is because COVID-19 has similar pathways of abnormal immune response mechanisms in its pathophysiology. Extrafollicular B cell activation can be found in critically ill COVID-19 patients, as seen in autoimmunity, and is strongly associated with poor prognosis in autoimmune diseases [55]. Peripheral blood B-cell subsets are markedly altered, with atypical memory B cells significantly expanded and classical memory B cells significantly decreased [56]. The results of the present study also confirmed the presence of different degrees of multiple immune infiltrates in RA and COVID-19 and the presence of each immune cell subtype in varying abundances. A significant negative correlation was observed between T cells CD4 memory resting and T cells CD8 in RA, whereas a significant negative correlation was observed between B cell memory and naive B cells in COVID-19, suggesting a potential association between these cells and the development of both diseases. For the identified key genes, although *LGMN* was positively and negatively associated with a variety of immune-infiltrating cells in COVID-19, it was not associated with immune-infiltrating cells in RA, and *NRGN* was positively associated with Macrophages M0 and Plasma cells and negatively associated with T cells CD4 memory-resting in both diseases, suggesting a significant negative association between B cell memory and naive B cells in COVID-19, suggesting a potential association between these cells and the development of the two diseases. We hypothesized that *NRGN* is associated with the development of immune infiltration in the presence of comorbidities. This suggests that immune infiltration analysis can help identify the differences and similarities between RA and COVID-19.

Therefore, we believe that *LGMN* and *NRGN* are involved in the development of RA and COVID-19 and can be valuable diagnostic markers, with the value of *NRGN* in immune infiltration likely to be higher. In addition, the associated regulatory networks, significantly involved pathways, and immune-infiltrating cells require further studies to explain their interactions and thus the potential to be therapeutic targets. However, this study has several limitations. First, the sample sizes used in the RA and COVID-19 datasets were relatively small, which may affect the generalizability and robustness of the findings. Second, although *LGMN* and *NRGN* were identified as potential diagnostic biomarkers shared between RA and COVID-19, their underlying regulatory mechanisms are unclear. No in vitro or in vivo experimental validation has been conducted to confirm their functional roles or diagnostic utility. Therefore, future studies involving larger, independent cohorts and experimental verification are necessary to further validate these candidate biomarkers and elucidate their mechanistic involvement in RA–COVID-19 comorbidity.

## Conclusions

By constructing a comorbidity model, we explored the potential molecular mechanisms shared by RA and COVID-19 and identified LGMN and NRGN as differentially expressed genes. These genes may act as key regulators in comorbid conditions and exhibit potential as clinical diagnostic biomarkers. The pathways in which the transcription factors associated with the diagnostic markers are significantly involved may become potential therapeutic avenues, and *NRGN* is closely associated with immune regulation in comorbidities.

## Supporting information

S1 Table Differential expressed genes RA patients in dataset. (XLSX)

S2 Table Differential expressed genes COVID-19 patients in dataset. (XLSX)

S3 Table GOKEGG enrichment analysis of differential expressed genes between RA and COVID-19 patients. (XLSX)

S4 Table Evaluation of the Diagnostic Performance of the two identified key genes using ROC Analysis. (XLSX)

S5 Table The proportion of 22 infiltrating immune cells in RA samples by CIBERSORT. (XLSX)

S6 Table The proportion of 22 infiltrating immune cells in COVID-19 samples by CIBERSORT. (XLSX)

S7 Table Drugability Assessment of Hub Genes. (XLSX)
